# Epigenetic Modifications and Head and Neck Cancer: Implications for Tumor Progression and Resistance to Therapy

**DOI:** 10.3390/ijms18071506

**Published:** 2017-07-12

**Authors:** Rogerio M. Castilho, Cristiane H. Squarize, Luciana O. Almeida

**Affiliations:** 1 Laboratory of Epithelial Biology, Department of Periodontics and Oral Medicine, School of Dentistry, University of Michigan, Ann Arbor, MI 48109-1078, USA; csquariz@umich.edu (C.H.S.); lubio2001@gmail.com (L.O.A.); 2 Comprehensive Cancer Center, University of Michigan, Ann Arbor, MI 48109-1078, USA

**Keywords:** epigenetics, HNSCC (Head and Neck Squamous Cell Carcinoma), DNA methylation, histone modifications, microRNA, cancer stem cell, chemoresistance, acetylation, histone H3

## Abstract

Head and neck squamous carcinoma (HNSCC) is the sixth most prevalent cancer and one of the most aggressive malignancies worldwide. Despite continuous efforts to identify molecular markers for early detection, and to develop efficient treatments, the overall survival and prognosis of HNSCC patients remain poor. Accumulated scientific evidences suggest that epigenetic alterations, including DNA methylation, histone covalent modifications, chromatin remodeling and non-coding RNAs, are frequently involved in oral carcinogenesis, tumor progression, and resistance to therapy. Epigenetic alterations occur in an unsystematic manner or as part of the aberrant transcriptional machinery, which promotes selective advantage to the tumor cells. Epigenetic modifications also contribute to cellular plasticity during tumor progression and to the formation of cancer stem cells (CSCs), a small subset of tumor cells with self-renewal ability. CSCs are involved in the development of intrinsic or acquired therapy resistance, and tumor recurrences or relapse. Therefore, the understanding and characterization of epigenetic modifications associated with head and neck carcinogenesis, and the prospective identification of epigenetic markers associated with CSCs, hold the promise for novel therapeutic strategies to fight tumors. In this review, we focus on the current knowledge on epigenetic modifications observed in HNSCC and emerging Epi-drugs capable of sensitizing HNSCC to therapy.

## 1. Introduction

Head and neck squamous cell carcinoma (HNSCC) is a heterogeneous group of tumors characterized by lesions in the oral cavity, larynx, pharynx (including nasopharynx, oropharynx, and hypopharynx), salivary glands, and thyroid [1,2,3]. HNSCC is the sixth most prevalent cancer worldwide, with approximately 780,000 new cases diagnosed each year and close to 350,000 deaths annually [4,5]. Despite several efforts to identify biomarkers for early detection and develop new treatments, the overall survival rate and prognosis remain poor [2,6,7]. The risk factors and carcinogens that participate in the development of HNSCC are well recognized [3,8]. Local recurrence and metastasis are limiting factors for the success of the treatment [9,10].

Over the years, cancer research efforts have focused on the genetic basis of tumor development and progression, identifying mutations and characterizing pathways that activate oncogenes and inactivate tumor suppressor genes. More recently, the research has pointed to epigenetic alterations as critic changes involved in the initiation and progression of human cancers [11]. Epigenetic alterations occur in an unsystematic way or as part of aberrant transcriptional machinery, promoting selective advantage to the tumor through the silencing of tumor suppressor genes or dysfunction in DNA repair genes [12,13]. Epigenetic modifications also contribute to the cellular plasticity during tumor progression, and the formation of tumor-initiating cells or cancer stem cells (CSCs) [14,15,16,17,18].

In this review, we discuss the current literature associated with the impact of epigenetic modifications on the progression and therapy of HNSCC, and examine aberrant DNA methylation, histone covalent modifications, chromatin remodeling, and non-coding-RNAs in HNSCC. We provide insights into recent studies on the epigenetics of CSCs, and novel treatment modalities using epigenetic drugs (Epi-drugs) alone, or in combination with conventional therapies.

## 2. Epigenetic Mechanisms in Cancer

The meaning of epigenetic was first introduced by Conrad Waddington in 1942 to define stable changes in the cell phenotype without genetic alterations [16,19]. Currently, epigenetic refers to stable and heritable changes in gene expression without changes in DNA sequence [20]. Epigenetic changes are fundamental mechanisms for carcinogenesis, and can serve as possible methods for early detection, treatment, and prognostic assessment for the cancer patients [21]. Epigenetic modifications include DNA methylation, histone covalent modifications, chromatin remodeling, and the effect of non-coding RNAs and polycomb proteins in gene expression [22,23]. Next, we will discuss the basis of epigenetics modifications.

### 2.1. DNA Methylation

The methylation of cytosine in CpG dinucleotides at the 5-carbon (5-methylcytosine) is a covalent modification of the DNA, and was the first and most explored chromatin modification [24,25,26]. CpG dinucleotides are highly accumulated in the 5′ promoter region of genes referred as CpG islands, which are unmethylated in transcriptionally active genes. Once CpG islands become methylated, transcriptional repression occurs [27].

A family of enzymes called DNA methyltransferases (DNMTs) controls methylation by catalyzing the transfer of the methyl group from *S*-adenosylmethionine to the cytosine. Three DNMTs (i.e., DNMT1, DNMT3a, and DNMTb) are fundamental to the methylation in mammals. DNMT1, called maintenance enzyme, is involved in restoring the parental DNA methylation profile after DNA replication, and exhibits a preference for hemimethylated DNA, ensuring the methylation status to the future cell generations [28,29,30]. DNA methylation in CpG dinucleotides, previously unmethylated or de novo DNA methylation, is carried out by DNMT3a and DNMT3b [12,26,30,31,32]. Moreover, DNA methylation works in association with chromatin modifications to repress gene expression [25]. DNA methylation provides a platform for many methyl-binding proteins, including MBD1, MBD2, MBD3, and MeCP2. These proteins operate by recruiting histone-modifying enzymes to coordinate chromatin dynamics [26,33].

Loss of DNA methylation was the first epigenetic alteration characterized in benign and malignant cancer cells [12,24,30]. Global DNA hypomethylation in cancer targets various genomic sequences, including repetitive elements, transposons, CpG dinucleotides in introns, and gene deserts, increasing genomic instability and activating proto-oncogenes [34,35]. In contrast, hypermethylation of the CpG islands on gene promoters contributes to the carcinogenesis through the silencing of tumor suppressor genes. For example, hypermethylation of *E-cadherin*, *pRB*, *p53*, and *CDKN2A* (p16^INK4a^/p14^ARF^), are common findings observed in cancer cell lines and primary tumors that result in gene silencing [36]. In fact, the use of next generation sequencing platforms has shown outstanding rates of abnormal CpG promoter methylation (5% to 10%) in various cancer types [25,26].

### 2.2. Covalent Histone Modification

The basic unit of the chromatin is the nucleosome, which consists of ~147 bp of DNA wrapped around a histone octamer containing two copies of four histone proteins (i.e., H2A, H2B, H3, and H4). Chromatin-modifying enzymes dynamically execute post-translational modifications (PTMs) of histones and DNA in a tightly regulated mechanism [26,37]. Histone PTMs are also an important mechanism that regulates chromatin structure and function [38]. Alterations in the patterns of histone PTMs are present in cancer at particular genes and global levels [37,39]. Histone tail projections from the octamer at the nucleosome undergo several post-translational covalent modifications involving the addition of chemical groups, such as methyl, acetyl, and phosphate. Less frequent alterations include ubiquitination, sumoylation, and ADP-ribosylation. These changes occur on the histone proteins at amino acid residues lysine, arginine, and serine [35,40].

Acetylation of lysine is the most important histone modification associated with transcription, chromatin architecture, and DNA repair. The addition of the acetyl group neutralizes the positive charge of the histone, weakening the electrostatic interaction between histones and the negatively charged DNA, promoting relaxation of the chromatin conformation favoring gene transcription [41,42,43]. Besides modification of histone charge, histone acetylation may also regulate intracellular pH. It is interesting that many tumors display low cellular pH and reduced levels of acetylated histones. Furthermore, the presence of low pH in tumors is also associated with poor prognosis for cancer patients [44]. In addition, histone acetylation has a role in recruitment of the general transcription machinery. In eukaryotes, general transcription is mediated by RNA polymerase II after the assembly of the preinitiation complex by the Transcription factor II D (TFIID). TFIID recognizes and selectively binds to sites with multiply acetylated histone H4 at the promoter [45]. Remarkably, the function of TFIID itself is regulated by TAFII250, which also has an acetyltransferase activity [46].

The addition of acetyl groups to lysine at the histone tails is catalyzed by enzymes called histone acetyltransferases (HATs), while histone deacetylases (HDACs) are responsible for removing acetyl groups. Although HATs are commonly driving gene expression, combined activation of HAT and HDAC is required for proper regulation of transcription [37,39]. HATs are classified into three major groups with nuclear location: (I) MOZ/YBF2/SAS2/TIP60, which belong to the MYST family; (II) GCN5 *N*-acetyltransferase or GNAT family; and (III) CBP/p300 family [47,48]. Different HATs work as both oncogenes and tumor suppressors, suggesting that a balanced acetylation is critical for cellular homeostasis [49]. HATs, such as the p300/(CREB binding protein) associated factor (PCAF), p300, and the CREB-binding Protein (CBP), target multiple non-histone proteins for acetylation, resulting in a gain of function for proteins such as p53 and BCL-6 [37,48]. For example, the HAT, Tip60, is involved in tumorigenesis through the modulation of ATM and the DNA damage response pathway, as well as the transcriptional activation of p53 and Myc. Down-regulation of Tip60 leads to genomic instability and impairment of the apoptotic signaling cascade, thereby helping the establishment of malignant transformation [50,51].

There are 18 human HDACs that are grouped into four classes according to the sequence homology to yeast. Class I (i.e., Rpd3-like enzymes) is comprised of HDAC1, 2, 3 and 8; and Class II (i.e., Hda1-like enzymes) is subdivided into IIa and IIb subclasses. HDAC4, 5, 7 and 9 belong to class IIa, and HDAC6 and 10 are in class IIb. Class III (i.e., Sir-like enzymes) consists of seven members called Sirtuins (SIRTs) which are NAD-dependent deacetylases. Class IV contains only HDAC11, which shares sequence homology with both HDACs class I and II. HDACs can regulate tumorigenesis through different mechanisms, such as downregulation of tumor suppressor genes and activation of oncogenic cell-signaling pathways [52]. HDACs possess low substrate specificity, and each enzyme can deacetylate multiple divergent histone sites. Although mutations in HDACs are not frequent, overexpression of HDACs is common in cancer [37,39,40]. HDACs, as well as HATs, act directly on non-histone proteins that are involved in tumor migration and metastasis. For example, HDAC2 deacetylates p53 and Cyclin-dependent kinase inhibitors 1B, 1C and 2A, which deregulates the apoptotic machinery, and disrupts the cell cycle [37,53,54].

Histone methylation in lysine, arginine, and histidine residues, does not alter the overall charge of the molecule, but notably, activates or represses gene transcription. The well-characterized sites of methylation occur on lysine residues, which can be mono-, di-, or tri-methylated. While histone methylases (HMT) add methyl groups, histone demethylases counteract this action by removing methyl groups from the histone tails [55]. Additionally, active genes typically display histone methylation markers such as H3K4me3, H3K36me3, and H3K79me3, while transcriptionally silenced genes frequently present repressive markers such as H3K27me3, H3K9me2, and H3K9me3 [56]. Often observed in several types of cancers, a member of HMT family responsible for the transcriptional (MLL1) displays loss of function and alters the expression of critical genes associated with cellular differentiation [57].

### 2.3. Chromatin Remodeling and Associated Proteins

Histone covalent modifications and DNA methylation are associated with a higher organization of the chromatin structure. The general process of inducing alterations in chromatin organization is named chromatin remodeling, which is an important mechanism for gene transcription. It requires modifications on the nucleosome structure to allow the transcription machinery to gain access to promoter regions [35]. Chromatin remodeler enzymes act through multiple mechanisms to overcome the barrier created by a densely-packed nucleosome. Four families of chromatin remodelers utilize energy derived from ATP hydrolysis to modify nucleosome organization, granting access to chromatin and target genes. They are the SWI/SNF family (switching defective/sucrose non-fermenting), the ISWI family(imitation switch), the NuRD family (Mi-2/nucleosome remodeling and histone deacetylation), and the INO80 family (inositol requiring 80). Deregulation of chromatin remodelers constitutes a common event in cancer development, progression, and therapeutic resistance [42]. Cancer-associated modifications of chromatin remodelers such as SNF5 (part of the subunit of the SWI/SNF chromatin remodeling complex), Brahma-related gene-1 (BRG1), and MTA family members (Metastasis-associated gene), are frequently found mutated in malignancies, suggesting a tumor suppressor role [58,59,60]. Overexpression of NuRD family members is often associated with the invasive potential of multiple cancers. The extension of modifications on chromatin remodeler enzymes becomes more evident with cancer genome sequencing data, which identified that ~20% of human tumors contain mutations in at least one member of the SWI/SNF complex [58,59,60,61,62]. Furthermore, loss of function of SNF5, a member of SWI/SNF family, results in enhanced susceptibility to multiple early childhood cancers, mediated by elevated expression of the polycomb gene *EZH2* (Enhancer of Zeste 2 Polycomb Repressive Complex 2 Subunit), resulting in increased H3K27me3 and cell cycle progression [63,64,65]. Conversely, RSF1 (Remodeling and Spacing Factor 1) gain of function is observed in a variety of human cancers, and is directly associated with tumor aggressiveness, poor therapeutic response, reduced survival, and poor prognosis [66,67,68].

### 2.4. Non-Coding RNA

In recent years, there is increased knowledge about non-coding ribonucleic acid (ncRNA), which goes above and beyond the well-known transfer RNA (tRNA) and ribosomal RNA (rRNA). Notably, a significant portion of the eukaryotic genome is transcribed into RNAs without protein- or peptide-coding function [69,70]. Many ncRNAs have several regulatory functions of mammalian organisms, particularly gene regulation at the levels of transcription, RNA processing, and translation. ncRNAs exploit the power of base pairing to selectively bind and directly act on other nucleic acids, but also act on epigenetic regulators. The majority of ncRNAs are involved in epigenetic regulations, mediating changes in chromatin conformation by directly targeting promoter regions, and consequently activating or repressing transcription [71]. ncRNA can be classified in microRNAs (miRNAs), PIWI-associated small RNAs (piRNAs) and long non-coding RNAs (lncRNAs), small interfering RNAs (siRNAs), enhancer RNAs (eRNAs), promoter-associated RNAs (PARs), among other RNAs, which play essential roles in regulating stem cells, cellular homeostasis, and contribute to the natural history of many human diseases, including cancer [72,73].

MicroRNAs (miRNAs) constitute the largest class of ncRNAs that regulate diverse cellular functions, including apoptosis, metabolism, cell growth, and differentiation. Deregulation in miRNAs expression changes normal cellular functions and is associated with the development of human diseases, including cancer [74,75,76]. miRNAs are comprised of 18–24 nucleotides, which usually act as post-transcriptional regulators of gene expression that recognize and bind to complementary sequences of target genes. miRNAs also inhibit protein synthesis via degradation of mRNA transcripts or repression of the translational machinery [77,78]. Deregulation of miRNA expression is found in cancer initiation and progression of most human cancers [72,75]. miR-21, for example, targets the tumor suppressor genes *PTEN* (Phosphatase and Tensin Homolog) and *PDCD4* (Programmed Cell Death 4) in several neoplasms [79,80]. The miRNAs can work as either tumor suppressors (e.g., miR-29b and miR-30) or display oncogenic characteristics (e.g., miR17-92 cluster) influencing cellular growth [74,81].

Interestingly, a subgroup of miRNAs also called epi-miRNA acts as target effectors of the epigenetic machinery, including DNMTs, HDACs, and polycomb genes, suggesting that miRNAs can indirectly regulate gene expression by interacting with epigenetic processes [35], for example, miR-29, that governs the expression of DNMT3a, 3b, and DNMT1 in lung cancer and acute myeloid leukemia, and miR-1, miR-140, and miR-449a, that regulate several HDACs in prostate cancer [71,74,82,83]. In contrast, several miRNAs are under epigenetic control in human cancers, due to its localization in CpG island regions, or epigenetic silencing mediated by promoter hypermethylation and histone modifications [84,85]. These facts expose the complexity of epigenetic changes in human carcinogenesis.

## 3. Epigenetic Modifications Associated with Tumor Progression and Drug Resistance in HNSCC

**Figure 1:**
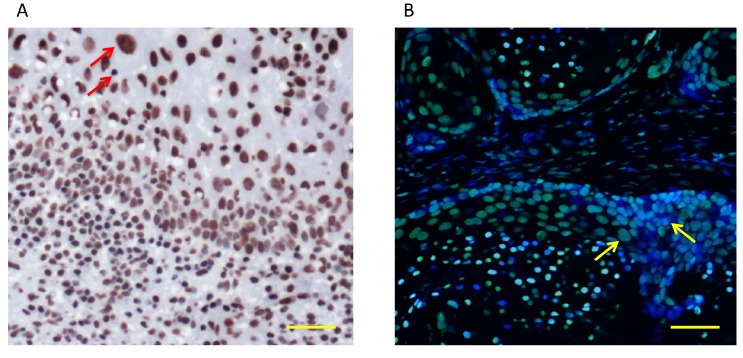
Changes in nuclear morphology may be associated with epigenetic modifications and deregulation of gene expression. (**A**) Immunocytochemistry of a head and neck squamous carcinoma (HNSCC) tumor sample stained for acetyl-H3 (Lys9). Note differences in the nuclear size and high levels of histone acetylation (red arrows, dark brown); (**B**) Immunofluorescence of an HNSCC tumor sample stained for acetyl-H3 (Lys9). Note differences in the nuclear size and protein accumulation depicted by yellow arrows. Scale bars represent 50 μm. Experimental procedure is described in [88].

**Figure 2:**
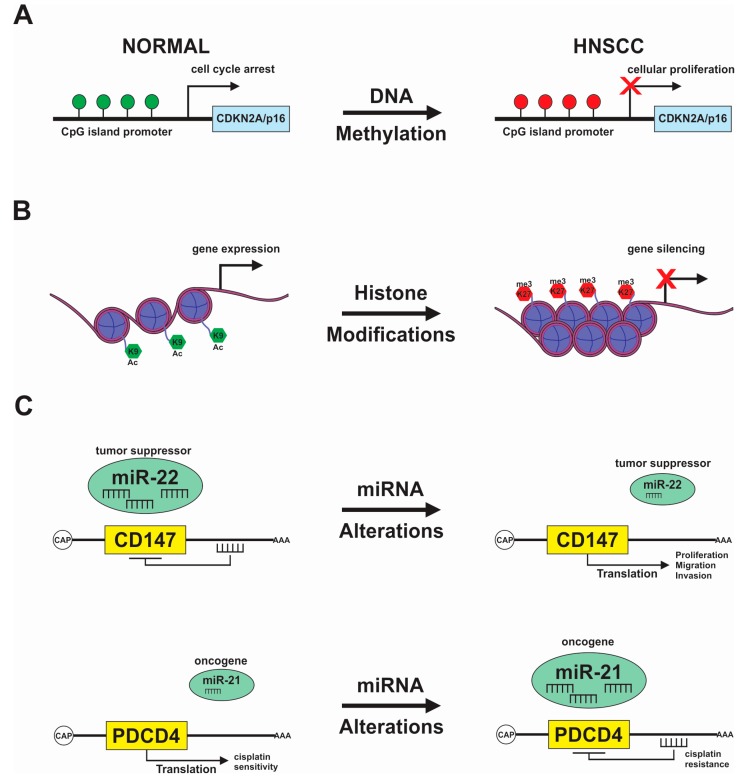
Overview of epigenetic alterations involved in the HNSCC progression and resistance to therapy. (**A**) DNA hypermethylation of the tumor suppressor *CDKN2A/p16^INK4a^* is the most frequent epigenetic modification observed in HNSCC, leading to cellular proliferation; (**B**) Changes in the chromatin structure lead to histone deacetylation and methylation of lysine in the histone tails resulting in gene silencing; (**C**) MicroRNAs work as a tumor suppressor and oncogenes regulate genes involved in HNSCC tumorigenesis. miR-22 acts as a tumor suppressor, controlling the levels of CD147 and it is frequently downregulated in HNSCC, increasing processes like proliferation, migration, and invasion (upper image). miR-21 is accumulated in HNSCC, showing oncogenic characteristics by targeting *PDCD4* gene increasing resistance to cisplatin treatment (lower image).

Despite recent advances in cellular and molecular biology techniques, the diagnosis of neoplasia is ultimately achieved by pathologists capable of identifying specific tissue characteristics and morphological features. Some of the microscopically-observed changes in tumor cells comprise alterations in the nuclear architecture and size (Figure 1). Indeed, changes in nuclear size, the presence of a condensed nuclear structure, prominent nucleoli, dense hyperchromatic chromatin, and a high nuclear-cytoplasmic ratio, are typical changes observed in HNSCC. These morphological changes suggest profound alterations in the chromatin structure and function of cancer cells [11]. Additionally, alterations in the nuclear morphology are predominantly associated with large-scale changes in gene expression mediated by the unbalance in global histone acetylation through redistribution of HDAC3 [86]. In HNSCC, reduction of nuclear size mediated by low levels of histone acetylation increased resistance to intercalating agents and reduced influx of DNA damage repair proteins to the nucleus [13]. Also, pharmacological inhibition of HDAC classes I and II decreased HNSCC proliferation and reduced the number of CSCs, a known subpopulation of cancer cells involved in tumor progression and the development of resistance to therapy [87]. The complexity of gene regulation by DNA methylation, histone modifications, chromatin remodeling and miRNAs are important mechanisms in tumor formation and progression; these mechanisms are currently active areas of research in head and neck cancer (Figure 2).

### 3.1. DNA Methylation Signature in HNSCC

Profiling DNA methylation constitutes a widely-applied tool to differentiate cancer from normal cells, as well as to identify subtypes of cancers, and to predict therapy outcome. Several studies have been carried out to explore the association between changes in DNA methylation and tumor progression of HNSCC. In fact, the DNA hypermethylation of tumor suppressor genes is an earlier event in carcinogenesis, and is responsible for uncontrolled cellular proliferation, which suggests that DNA hypermethylation is a potential marker for early diagnosis of HNSCC [89].

Over the years, DNA hypermethylation has been the most assessed epigenetic modification in HNSCC; however, the presence of global hypomethylation is a common finding during the tobacco-associated development of oral squamous cell carcinomas (OSCC) [90]. Methylation profiles analyzed in oral rinse revealed hypomethylation of *LINE-1* (group member of the long interspersed nuclear elements family—LINEs) independent of the tumor grade, site, or risk factors, which suggests the potential use of LINE-1 as a biomarker for early detection of OSCC [77,91]. Hypomethylation of individual genes, such as the *WISP1* gene (WNT-inducible-signaling pathway protein 1), was identified in OSCC associated with lymph node metastasis, suggesting its use as a potential diagnostic marker [92]. Survivin is a well-known protein related to tumor progression, and responsible for increased cellular proliferation and reduced apoptosis. Notably, promoter hypomethylation of *BIRC5* (the gene that encodes surviving) is frequently found in OSCC, and leads to a more aggressive and invasive tumor phenotype [77,93]. DNA hypomethylation may also be involved in anticancer drug resistance, which results in accumulation of the ATP-binding cassette subfamily B member 1 (ABCB1), and cisplatin resistance in OSCC [94,95]. Furthermore, hypermethylation of CpG islands in the promoter region is associated with HNSCC formation and progression. Some examples of the frequently methylated genes in HNSCC are shown in Table 1 [96,97].

Several studies show that *p16^INK4a^* (*p14^ARF^*) is one of the most hypermethylated genes in HNSCC. *p16^INK4a^* is a tumor suppressor gene encoded by the CDKN2A locus [5,77,98,99,100,101,102,105] The p16 function limits G1 cell cycle progression by inhibiting the cyclin-dependent kinases CDK4 and CDK6, and consequently compromises control of cellular proliferation, in addition to increased angiogenesis [103]. Several studies have suggested that *p16^INK4a^* hypermethylation could be used as a biomarker in the prediction of malignant transformation, due to the methylation-associated metastasis and poor survival in HNSCC [77]. Inactivation of *p15^INK4b^* (CDKN2B) associated with *p16^INK4a^* hypermethylation observed in precancerous oral tissues, suggests that methylation of these genes constitute early events in oral pathogenesis [99,104].

Hypermethylation of E-cadherin and DNA repair genes are also frequent events associated with tumor progression and invasion. Silencing of E-cadherin leads to the deregulation of essential cell functions, like adhesion, polarity, and morphogenesis, which results in increased motility and aggressive behavior of OSCC cells [5,99]. Hypermethylation of DNA repair genes such as *MGMT* (06-methylguanine-DNA methyltransferase), a DNA repair enzyme responsible for removing adducts from the DNA, results in reduced apoptosis and increased resistance of cancer cells to alkylating agents [5].

One of the most appealing areas of research in epigenetic modifications is the discovery and characterization of biomarkers. Epigenetic biomarkers have the potential to serve as markers for early stages of cancer to the identification of tumors with higher chances to develop resistance to therapy, or with great odds to produce local and distant metastasis. Discovery of biomarkers using samples of body fluids has been explored with the intent to improve screening accuracy and cost-effectiveness and decrease invasiveness. For head and neck cancer, saliva and oral rinse have been used to obtain DNA from the oral epithelium to analyze the DNA hypermethylation profile [101,108] Among potential biomarkers for oral carcinogenesis, *HOXA9* (Homeobox A9) and *NID2* (Nidogen 2) hold high promise due to their apparent sensitivity and specificity. Additional methylated genes, such as *HS3ST2*, *NPY*, *EYA4*, *WT1*, and the combination of *E-cadherin*, *TMEFF2*, and *MGMT* methylations, could also serve as potential biomarkers for early detection of HNSCC [78,99,107]. *CHFR* (i.e., Checkpoint with FHA and RING finger domains) hypermethylation is a late event in HNSCC, suggesting an association with tumor progression and potential use as a biomarker of late stage disease progression [99]. The identification of epigenetic biomarkers is paving the road to more attractive and reliable screenings to identify the development of HNSCC and better characterize subtypes of tumors to help implement personalized treatments [77].

Interestingly, few signaling pathways seem to be particularly vulnerable to DNA methylation in cancers. This is the case of the WNT molecular signaling, which is responsible for regulating the transcription of β-catenin, a key molecule involved in the carcinogenesis process, the proliferation of tumor cells, and the promotion of tumor survival. In oral cancer, several genes related to WNT signaling are found silenced by DNA methylation, including *SFRP* (Secreted frizzled-related protein), *SOX17* (SRY-box 17), and *WIF1* (WNT inhibitory factor 1) [100]. WNT signaling is a well-characterized pathway activated in embryogenesis and the maintenance of stem cells. Likewise, aberrant activation of WNT signaling is observed in cancers and leads to the accumulation of cancer stem cells, which is involved in the tumor progression, metastasis, and resistance to therapy [106,109].

### 3.2. Histone Acetylation and Chromatin Modifications in HNSCC

The dynamic mechanism of core histones is mediated by several posttranslational modifications that take place on histone tails as lysine acetylation. Histone acetylation is highly controlled by a balance between HATs and HDACs [39]. Given that histone modifications drive the chromatin organization and gene expression, it is no surprise that abnormal alterations in histone acetylation are associated with cancer development [52]. Chromatin is divided into two distinct conformations: heterochromatin, which is densely compacted and transcriptionally silenced, and euchromatin, which is less condensed and transcriptionally active. The structural status of the chromatin ultimately results in the transcriptional position of the gene [48].

The identification of a vast number of acetylation sites has been challenging because of the reversible nature and low abundance of this modification [110]; therefore, few studies described alterations in histone acetylation at specific lysine residues in HNSCC. Our group identified loss of histone H3K9ac as a marker for chemoresistance, associated with NFκB signaling [13] and accumulation of CSC [88,111]. We also showed that reduction of H3K9ac is associated with increased cell proliferation and activation of epithelial-mesenchyme transition (EMT) during oral carcinogenesis, suggesting the involvement of H3K9ac in tumor progression of HNSCC [87,112]. Furthermore, low levels of H3K4ac and high levels of H3K18ac are associated with OSCC progression and poor prognosis [113].

Changes in the expression of several HDACs were reported in multiple cancers and associated with the regulation of cell cycle-associated genes, cellular differentiation, apoptosis, angiogenesis, invasion, and migration [114,115,116]. For instance, HDAC6 is overexpressed in advanced stages of HNSCC, suggesting that its activity may be important in determining tumor aggressiveness in oral cancers [117]. HDAC6 can deacetylate α-tubulin increasing cell motility, a fundamental process in the development of tumor metastasis [77]. HDAC2 is overexpressed in OSCC that results in enhanced stability of the protein HIF-1α, which leads to increased invasion and migration of HNSCC [77,118]. HDAC7 is also overexpressed in head and neck cancer, and its accumulation activates c-MYC, promoting cellular proliferation [119]. Accumulation of HDAC8 induces proliferation by inhibiting activation of caspases and autophagy in OSCC [120]. HDAC9 has an oncogenic role in OSCC, increasing tumor growth by targeting pro-apoptotic genes, and its overexpression was correlated with reduced overall survival [114].

The SIRT family (Sirtuins) belongs to class III of NAD^+^-dependent histone deacetylases, which are implicated in the cell cycle, transcription regulation, and metabolism. SIRTs play opposite roles in cancer, acting as a tumor suppressor in some cancers, protecting against DNA damage and oxidative stress, and promoting cancer in others [121,122]. SIRT1, 2, 3, 5 and 7 are diminished in advanced stages of HNSCC and can be potential prognostic markers [123], while SIRT6 is accumulated in peripheral blood of patients with HNSCC [124].

Chromatin remodelers are important molecules responsible for controlling chromatin accessibility and nucleosome positioning in response to stimuli for DNA-driven biological processes. Disruption of chromatin remodelers is intimately implicated in cancer development, progression and therapeutic resistance, due to the impact on deregulated gene transcription machinery. Given these functions, a better insight into chromatin remodelers will provide the basis for the development of new anticancer therapeutic strategies [42]. ACTL6A is a subunit of SWI/SNF complex of chromatin remodeling. In HNSCC, ACTL6A is highly expressed, working as an oncogene changing chromatin organization of key genes, and is associated with loss of cellular differentiation and increasing of proliferation [125]. ING4 belong to ING family of chromatin remodelers, and has been associated with induction of apoptosis, cell cycle arrest, tumor growth, and angiogenesis [126,127,128]. ING4 is found either in the nucleus or the cytoplasmic in HNSCC. Increased levels of cytoplasmic ING4 are often involved in malignant progression, whereas nuclear ING4 may modulate the transactivation of target genes, promoting apoptosis and cell cycle arrest [129]. Another chromatin remodeler frequently deregulated in cancer is RSF-1, a member of ISWI family found up-regulated in OSCC, which displays increased tumor invasion, lymph node metastasis, and advanced tumor stages. RSF-1 also potentiates resistance to radiotherapy and chemotherapy through remodeling the chromatin of target genes [130].

Alterations in chromatin remodelers are not only important in the carcinogenesis process but also in the development of resistance to therapy. BRG1 and BRM are catalytic components of SWI/SNF, which mediate resistance to cisplatin in HNSCC. MTA1 overexpression induces cisplatin resistance in nasopharyngeal carcinoma by promoting cancer stem cell accumulation in a PI3K/AKT-dependent manner [131,132]. MTA1 is an important chromatin modifier that is upregulated in HNSCC. MTA1 regulates E-cadherin, and the WNT/β-catenin pathway activates EMT, favoring tumor invasion [133].

### 3.3. Non-Coding RNAs in HNSCC

MicroRNAs are a class of non-coding RNAs that is well investigated in HNSCC. Similar to proteins, miRNA dysfunction can be driven by alterations in miRNA expression resulting from gene mutations, epigenetic modifications, or deficiency in the miRNA processing. Genetic and epigenetic alterations in several miRNAs were correlated with cancer [74]. In OSCC, miR-26, -34b, -137, -193a and -203 are found downregulated via CpG hypermethylation [134]. Interestingly, miR-26a targets DNMT3B enzyme, stimulating cell proliferation and demonstrating the intimate association between different epigenetic mechanisms in oral carcinogenesis [135,136].

MicroRNAs can work as either tumor suppressor or oncogene to influence cellular growth [81]. For example, overexpression of miR-21 works as an oncogene when targeting *PDCD4* gene, resulting in increased incidence of metastasis and enhanced invasive potential in OSCC [137]. miR-184 promotes the accumulation of c-MYC, inducing cell proliferation and overexpression of miR-24, that targets CDKN1B and decreases its levels promoting cell proliferation in oral cancer [138]. miR-211 is frequently overexpressed in cancer, and associated with tumor progression, nodal metastasis, vascular invasion, and poor prognosis of oral carcinoma [77].

In contrast, many miRNAs that play a role as tumor suppressors are often found downregulated in HNSCC. For example, miR-7 suppresses tumorigenesis by targeting the IGFR/AKT pathway and restricts cellular proliferation [139]. Similarly, downregulation of miR-22 results in the promotion of cell proliferation and increases tumor migration mediated by the overexpression of CD147, which suggests that miR-22 displays a tumor suppressor activity [140]. Other examples of reduced levels of miRNA leading to aggressive tumor behavior includes miR-195, which results in increased cell proliferation by targeting cyclin D and Bcl-2 [136], miR-222 and miR-34a, that results in increased MMP production in tongue squamous cell carcinoma [136,138], and miR-138, that results in enhanced migration and invasion through the accumulation of RhoC and ROCK2, and is also a potential therapeutic target for patients with oral cancer [139]. Moreover, miR-219 expression remarkably suppresses cell proliferation, colony formation, migration, and invasion by targeting PRKCI in HNSCC, suggesting that miRNAs play a significant role in tumor suppression [138].

Over 10% of all characterized miRNAs are associated with a chemoresistance phenotype by affecting central mechanisms involved in the development of cancer chemoresistance phenotypes, such as drug efflux, apoptotic dysfunction, cellular proliferation, and DNA damage repair [82,141,142,143,144,145,146,147]. miRNAs are also correlated with chemoresistance of HNSCC cells (Table 2). In HNSCC, increased levels of miR-21 are associated with low levels of PTEN and TPM1, as well as PDCD4. Accumulation of miR-21 has been widely observed in several cancers, and is related to its oncogenic function and role in resistance to cisplatin treatment [80,83]. miR-23a and -214 are also associated with chemoresistance. In oral cancer, both miRNAs are overexpressed, attenuating resistance to cisplatin by upregulating TOP2B [138].

## 4. Epigenetic Modifications of Cancer Stem Cells

The cancer stem cells (CSCs) model implicates that each tumor consists of a heterogeneous population of tumor cells and a small subpopulation of cancer cells endowed with stem cell properties and the ability to generate new tumors and metastasize. CSCs are undifferentiated, pluripotent, and have the ability of self-renewal, giving rise to other malignant daughter cells. Tumor cells are usually responsive to conventional chemotherapy, whereas CSCs are resistant to therapy, and will ultimately re-establish the tumor at the local or distant sites [35,148,155,156] Therefore, a better understanding of the regulatory mechanisms that control the population of CSCs is essential to the development of more efficient therapeutic strategies against HNSCC.

Despite the uncertain origin of CSCs, it is widely accepted that CSCs share key characteristics of embryonic stem cells, that include the capacity for self-renewal and the maintenance of an undifferentiated phenotype. CSCs can block differentiation by suppressing gene expression through epigenetic reprogramming, altering the profile of histone modifications and DNA methylation, similar to what is seen in embryonic stem cells [149]. Proteins from the polycomb group (PcG) are involved in the epigenetic regulation of gene expression, and play a fundamental role in the maintenance and differentiation of stem cells. EZH2 is a histone methyltransferase that works as a subunit of PcG. EZH2 catalyzes the trimethylation of lysine 27 on histone H3 (H3K27me3), regulating the expression of genes that determine the balance between cell differentiation and proliferation. Overexpression of EZH2 promotes self-renewal, prevents stem cells differentiation, and is associated with aggressiveness and poor prognosis in oral cancer [105,149].

BMI1 is a member of the polycomb group, which promotes chromatin silencing. BMI1 is required for the maintenance of adult stem cells, and is involved in carcinogenesis of different cancers, and may be a marker for cancer stem cells in HNSCC [150,151]. Wnt/β-catenin pathway has important functions in the self-renewal and differentiation programs of CSC. Besides promoter hypermethylation, deregulation of the Wnt/β-catenin pathway is also mediated by histone modifications [18,152]. Epigenetic mechanisms also upregulate several genes associated with Notch and Hedgehog signaling during CSC accumulation, self-renewal, and differentiation in carcinogenesis. Aberrant epigenetic modifications may deregulate a complex signaling process that modulates normal stem cells activities during tumor formation. These alterations ultimately contribute to CSC proliferation and maintenance, as well as tumor progression and invasion. Therefore, epigenetic regulation of key pathways may work as potential targets for therapy against CSCs [18].

CSCs tend to be more resistant to chemo and radiotherapy compared to non-CSC tumor cells, due to specific mechanisms like increased expression of drug efflux transporters [153,154,157,158], decreased expression of drug uptake transporters [159], increased drug detoxification [160], prolonged cell cycle arrest [161], increased DNA damage repair [162], activation of EMT [163,164], and epigenetic reprogramming [148]. Of note, our group and others have shown that treatment with cisplatin, the most common chemotherapeutic agent against head and neck cancer, promotes accumulation of CSCs, probably through mechanisms involving loss of global histone acetylation and epigenetic reprogramming [88,111,165].

Multidrug resistance refers to the ability of cancer cells to resist different anticancer drugs, which is one of the major clinical obstacles in cancer chemotherapy. Multidrug resistance can be caused by several mechanisms, including an increase of efflux transporters [166]. CSCs display high expression of drug efflux pumps, such as ATP-binding cassette (ABC) transporter family proteins, which are important for efflux of drugs across the plasma membrane [155,167]. Elevated levels of *ABCG2* is a marker to identify CSCs in oral cancer [168]. Interestingly, *ABCG2* is epigenetically regulated by miR-222 in tongue squamous cell carcinoma, and deregulation of the miR-222–*ABCG2* contributes to cisplatin resistance, and cancer aggressiveness [169]. Also, *ABCB1* and *ABCG2* are expressed simultaneously in many cancer cells, including oral cancer [166]. They are found hypomethylated and associated with chemoresistance in solid and hematological malignances [124].

## 5. Epigenetic Drugs and HNSCC Therapy

One of the fundamental goals of cancer research is to translate new findings to a clinically relevant setting. A better understanding of the mechanisms associated with epigenetic modifications will result in the development of new epigenetic markers capable of early detection of tumors and maintenance of continuous surveillance. Also, the identification of distinct epigenetic profiles will corroborate to the identification of prognostic tools and a potential predictor of tumor response to therapy [104]. As we move towards personalized and precise medicine, it is important to remember that the genetic material is identical in every cell, while epigenetics is highly variable within different cells and tissues of an organism, and is also affected by aging and environmental factors [99].

Given the reversible nature of epigenetic modifications, it is clear that changes observed during disease progression become attractive targets for cancer therapy. An increasing number of epigenetic drugs are in development. Currently, they are classified into two groups: histone deacetylase inhibitors (HDACi), and DNA methyltransferase inhibitors (DNMTi). We will certainly see an increasing number of pharmacological classes as our knowledge on epigenetics evolves. Six US Food and Drug Administration (FDA)-approved epigenetic agents are applied in the clinic: two DNMTi, 5-azacytidine (i.e., Vidaza) and 5-aza-2′-deoxycytidine (i.e., Decitabine), and four HDACi, suberoylanilide hydroxamic acid (i.e., Vorinostat), F-228 (i.e., Romidepsin), LAQ-824 (i.e., Farydak), and LBH-589 (i.e., Panobinostat). Many others agents are in the pipeline [105,170]. Results from basic science and clinical research data are starting to pile up. Use of epigenetic inhibitors like the DNMTi, Zebularine, led to tumor growth inhibition, as evidenced by decreased of proliferation and cell cycle arrest in HNSCC cell lines [77]. The histone methylation inhibitor 3-deazaneplanocin (DZNep) reversed the aggressive characteristics of oral carcinoma cells through the dynamic regulation of epithelial plasticity and blocking EMT via the reprogramming of gene expression profile [171].

DNA hypermethylation is a common event in HNSCC that leads to gene transcription silencing of cell cycle controlling genes. Thus, DNA methyltransferases, the enzymes driving methylation dynamics, are important targets for cancer treatment [172,173]. The reversal of DNA hypermethylation by pharmaceutical agents like 5-azacytidine, a DNA methyltransferase inhibitor, constitutes an effective treatment of several cancers, including HNSCC. 5-Azacytidine, as single treatment, inhibited the proliferation of HNSCC tumor xenografts, however, it failed to reduce the severity of the lesions. In contrast, the association of 5-azacytidine with retinoic acid was efficient in reducing the effects of chemical carcinogenesis [174,175]. Alternatively, demethylation agents can also be administered as chemosensitizer agents against cancer. Administration of decitabine shows promising results in sensitizing HNSCC cell lines to cisplatin, reducing overall dose requirements for cisplatin-induced apoptosis. Combination treatment of cisplatin and decitabine significantly reduced HNSCC growth [176]. The DNMTi zebularine also works as an adjuvant drug to cisplatin and 5-fluoro-uracil, increasing apoptosis in HNSCC [77,177].

HDACi represents a growing class of anticancer agents that regulates gene expression through histone acetylation modulation. In several cancers, treatment with HDACi results in differentiation, cell cycle arrest, apoptosis, and decreasing of angiogenesis [26,178,179]. Through hyperacetylation of histone and non-histone targets, HDACi enables the reestablishment of cellular acetylation homeostasis, and restores normal expression and function of several proteins that may reverse cancer initiation, progression, and therapy resistance [52].

Although HDACi has been successfully used in the treatment of hematological malignancies, it shows limited clinical activity as a single agent in solid tumors. The most promising HDACi results were seen when it was combined with other therapeutic regimens, including its combination with radio and chemotherapy, which resulted in synergistic or additive effects. Indeed, HDACi is mostly effective against HNSCC when administrated with other therapeutic agents [178,180,181]. Treatment using combined Trichostatin A (TSA) and all-trans-retinoic acid (ATRA), synergistically increased the growth-inhibitory effect, and strongly induced transcriptional activation of target genes, which restored tumor sensitivity to retinoic acid in HNSCC cell lines [182]. Association of TSA and proteasome inhibitor PS-341 (Bortezomib) was also investigated in HNSCC. Although TSA alone did not induce apoptosis, it significantly enhanced PS-341-induced apoptosis through activation of caspase [1]. MS-275, a synthetic benzamide HDACi, also increased the cytotoxic effect of cisplatin in the treatment of OSCC [183].

Combined regimens seem to provide a better clinical outcome to HNSCC patients. An example of this strategy is the co-administration of HDACi and cisplatin in HNSCC, which displays synergistic effects in inducing greater cytotoxicity and apoptosis, compared to cisplatin alone [5,77,177,183,184]. Co-administration of HDACi and cisplatin also resulted in the activation of the miR-107 and miR-138 followed by the disruption of cellular migration and invasion of HNSCC [185]. Additionally, administration of TSA reversed chemoresistance to cisplatin in HNSCC cell lines by targeting NFκB nuclear accumulation [13].

In the search for novel therapeutic strategies capable of improving the efficacy of tumor treatment while limiting toxicity, a combination of radiotherapy with epigenetic drugs seems to be a valuable option. Preclinical radiosensitization using HDACi has been reported as a feasible strategy for solid tumors, including HNSCC. TSA, SAHA (suberoylanilide hydroxamic acid; Vorinostat), M344 (an analog of SAHA), and depsipeptide (FR90228), modulate cellular responses to ionizing radiation, and promote cell cycle arrest and apoptosis in HNSCC [186]. Additionally, demethylating agents, such as 5-aza-2′-deoxycytidine, target DNMTs, resulting in global demethylation, and are also radiosensitizing agents for HNSCC treatment [187,188].

As epigenetic mechanisms have important functions in modulating stem cell properties in cancer cells, targeting components of these epigenetic pathways would help to eradicate the population of CSCs and non-CSCs. Recent studies suggest that small doses of the DNMTi 5-aza-2′-deoxycytidine are sufficient to reduce the tumorigenicity by targeting CSCs [189,190]. We have recently found that changes in chromatin acetylation of HNSCC unexpectedly triggered the reduction of CSCs. Hyperacetylation of chromatin in HNSCC cells using TSA disrupted the ability of CSCs to generate and maintain tumor spheres. We also found that TSA reduces the enzymatic activity levels of ALDH, a well-stablished marker of CSCs [87]. HDAC inhibitors SAHA and TSA, also decreased cancer stem cell markers CD44 and ABCG2, and genes related with cellular stemness, as well as the EMT phenotype in oral carcinoma [191]. Likewise, low levels of SAHA also abolished the CSC population in salivary gland tumors [88,111].

## 6. Conclusions

Increasing knowledge on epigenetic modifications is contributing to the overall understanding of cancer biology. Epigenetic changes may be used as tools to diagnose, treat, and provide prognostic information for HNSCC patients. The clinical importance of research efforts to identify and validate novel epigenetic modifications associated with cancer chemoresistance cannot be underestimated. Discovery of new epigenetic biomarkers for individual cancer chemoresistance can open the possibility for the development of novel drugs (Epi-drugs), which can be used as an adjuvant therapy associated with conventional chemotherapeutic drugs, enhancing tumor sensitivity to traditional agents and ultimately increasing treatment effectiveness. The emerging understanding of the biological effects of HDACi over the population of CSCs is a breakthrough on epigenetic studies. The success of deploying HDACi as sensitizer agents before the administration of chemotherapeutic agents has underscored the importance of targeting CSCs using Epi-drugs for HNSCC and salivary tumors. Therefore, epigenetic modifications induced by Epi-drugs, aligned with the prospective identification of epigenetic biomarkers, are an exciting frontier in tumor biology waiting to be explored.

## Figures and Tables

**Table 1:** List of the frequently altered genes by DNA methylation in HNSCC. N/A: not applicable.

Gene ID	HGNC ID	Function	Methylation Status	References
CDKN2A (p16; p14^ARF^)	HGNC:1787	cell cycle arrest/apoptosis/senescence	hypermethylated	[5,77,89,98,99,100,101,102,103,104]
MGMT	HGNC:7059	DNA damage repair	hypermethylated	[5,77,89,98,100,101,102,103,104]
DAPK	HGNC:2674	apoptosis	hypermethylated	[5,89,98,100,101,102,103,104]
APC	HGNC:583	cellular adhesion/migration	hypermethylated	[5,77,98,100,101,102]
RASSF1	HGNC:9882	cell cycle arrest/ cytoskeleton organization	hypermethylated	[5,77,89,101,103,104]
CDH1	HGNC:1748	cellular adhesion	hypermethylated	[77,100,102,103,104]
HOXA9	HGNC:5109	cell differentiation	hypermethylated	[91,98,105,106]
MLH1	HGNC:7127	DNA damage repair	hypermethylated	[5,77,101]
CDKN2B (p15)	HGNC:1788	cell cycle arrest	hypermethylated	[5,77,100,101]
TIMP3	HGNC:11822	extracellular matrix degradation	hypermethylated	[89,98,103,104]
ATM	HGNC:795	DNA damage repair	hypermethylated	[5,107]
MINT31	N/A	chromatin remodeling	hypermethylated	[89,98,100]
CALCA	HGNC:1437	cellular metabolism/ inflammatory response	hypermethylated	[99,103,106]
NPY	HGNC:7955	cell proliferation	hypermethylated	[105,106]
HS3ST2	HGNC:5195	circadian rhythm	hypermethylated	[105,106]
ADGRE3 (EMR3)	HGNC:23647	cellular surface receptor /inflammatory response	hypomethylated	[105]
PI3	HGNC:8947	inflammatory response	hypomethylated	[105]
AIM2	HGNC:357	apoptosis/ inflammatory response	hypomethylated	[105]
SPP1	HGNC:11255	cellular adhesion/ inflammatory response	hypomethylated	[105]

**Table 2:** Deregulation of miRNAs associated with chemoresistance in HNSCC.

ID	Expression	Drug Resistance	Reference
miR-21	increased	cisplatin	[148]
miR-634	increased	paclitaxel	[149]
miR-181a	increased	cisplatin	[150]
miR-23a	increased	cisplatin	[151]
miR-10b	increased	cisplatin	[152]
miR-98	increased	doxorubicin	[153]
miR-214	increased	cisplatin	[140]
miR-200b	decreased	cisplatin	[154]
miR-15b	decreased	cisplatin	[154]
